# DEMOGRAPHIC CORRELATES OF AGING-RELATED GENE EXPRESSION LEVELS AMONG OLDER AMERICANS

**DOI:** 10.1093/geroni/igad104.0720

**Published:** 2023-12-21

**Authors:** Qiao Wu, Eric Klopack, Jung Ki Kim, Jessica Faul, Bharat Thyagarajan, Steve Cole, Eileen Crimmins

**Affiliations:** University of Southern California, Los Angeles, California, United States; University of Southern California, Los Angeles, California, United States; University of Southern California, Los Angeles, California, United States; University of Michigan, Ann Arbor, Michigan, United States; University of Minnesota, Minneapolis, Minnesota, United States; University of California Los Angeles, Los Angeles, California, United States; University of Southern California, Los Angeles, California, United States

## Abstract

Measures of conserved transcriptional response to adversity (CTRA), DNA damage response (DDR), and senescence-associated secretory phenotype (SASP) gene expression can be regulated by social and environmental exposures. The resulting synthesis of proteins can affect aging. To further understand how demographic disparities get “under the skin” and link to health outcomes, we examine associations between (A) age, gender, and race/ethnicity and (B) three gene expression composite scores derived from RNA sequencing data in a nationally representative sample of 2,680 Americans aged 55 and older from the 2016 Health and Retirement Study. We also compare the associations across models using multiple approaches to controlling for blood cell composition. OLS regressions suggest that compared to those aged 55-64, being older is associated with a lower inflammatory sub-score of CTRA (Ages 75-84: β=-0.04, p=0.008; Ages 85+: β=-0.12, p<0.001), lower CTRA (Ages 85+: β=-0.15, p<0.001), higher DDR (Ages 85+: β=0.03, p=0.042), and lower SASP (Ages 75-84: β=-0.05, p=0.015; Ages 85+: β=-0.07, p=0.017) scores. Women have significantly higher inflammatory (β=0.06, p<0.001) and antiviral (β=0.04, p=0.002) sub-scores of CTRA. Compared to non-Hispanic Whites, non-Hispanic Blacks have a higher inflammatory sub-score of CTRA (β=0.04, p=0.026) and a lower SASP score (β=-0.19, p<0.001), and Hispanics have a lower SASP score (β=-0.08, p<0.001). Adjusting for education, drinking, smoking, BMI, and chronic conditions does not change the results much. Overall, using expression levels of the genes that mark major leukocyte subsets to control for blood cell composition does not notably differ from approaches based on complete blood count and flow cytometry.

